# Design and Characterization of Gypsum Mortars Dosed with Polyurethane Foam Waste PFW

**DOI:** 10.3390/ma13071497

**Published:** 2020-03-25

**Authors:** Isabel Santamaría Vicario, Lourdes Alameda Cuenca-Romero, Sara Gutiérrez González, Verónica Calderón Carpintero, Ángel Rodríguez Saiz

**Affiliations:** Department of Construction, University of Burgos, Calle Villadiego s/n, 09001 Burgos, Spain; isantamaria@ubu.es (I.S.V.); lalameda@ubu.es (L.A.C.-R.); sggonzalez@ubu.es (S.G.G.); vcalderon@ubu.es (V.C.C.)

**Keywords:** recycled material, sustainable buildings, gypsum mortar, polyurethane foam waste (PFW)

## Abstract

The properties and the behaviour of plaster mortars designed with Polyurethane Foam Waste (PFW) are studied in this investigation. A characterization of the mixtures is completed, in accordance with the technical specifications of European Norms. The incorporation of polyurethane waste foam can yield porous and lighter mortars, with better resistance to water-vapour permeability, although with weaker mechanical strength and higher levels of absorbency. Nevertheless, suitable mechanical strengths were achieved, resulting in a new material that is compliant with the requirements of the construction industry. The use of PFW in the the manufacture of gypsum mortars for construction reduces the consumption of natural resources and, at the same time, recovers an industrial waste that is otherwise difficult to recycle.

## 1. Introduction

The degree of development of our society has brought with it important levels of wellbeing for the population, although social inequalities unbefitting a civilized world still persist. Development has also provoked considerable negative effects, both for the environment and for the quality of life of many people. One of the most significant is industrial waste, with a particular impact on the physical deterioration of the environment and the global warming of our planet [1,2,3,4].

Concerns are growing over the efficient management of the waste generated in industrial processes [5,6,7,8,9]. The Waste Framework Directive 2008/98/EU of the European Union established measures to mitigate the environmental impact of waste generated by human activity through its management, recycling, reuse and whenever possible, through its recovery, establishing 2020 as the target for the achievement of those objectives [10].

Polyurethane is a basic material that is used in numerous industrial applications, such as the manufacture of foam for the automobile industry, the manufacture of packaging, the furniture industry, and as insulative materials. These activities generate significant volumes of polyurethane waste with other materials that, in most cases, are only managed through dumping in landfill sites. This sort of waste may be characterized by its low biodegradability and because its molecular structures persist over time, and are difficult to transform into simpler and more stable shapes [11,12,13]. In addition, as polyurethane has a very diverse chemistry, it is difficult to find a single method for its recycling, for which reason it is necessary to seek alternative methods for its recovery [14,15,16].

One of the direct uses of Polyurethane Foam Waste (PFW) is its recovery for the production of energy through its incineration in industrial furnaces. It is also used to obtain compound foams from panel-cutting waste, which, once shredded, are added to chemical binders such as diphenylmethane diisocyanate (MDI) or polymethylene polyphenyl isocyanate (PAPI) [17]. Nevertheless, there are, at present, numerous investigations seeking alternatives for PFW, promoting its recovery and recycling as a raw material for other industrial processes. In the field of the Horizon 2020 European Projects, the *PUReSmart* project is investigating the use of both rigid and flexible PFW for the design and manufacture of new polymetric materials [18]. In the construction field, the *Life Repolyuse* project is investigating the use of rigid polyurethane foam in the design of new construction materials for the automobile industry and refrigeration industry in [19].

In Spain, there are various Research Projects with investigations into development to recover PFW. The *REPUR* project is investigating chemical and mechanical recycling processes for their recovery in the manufacture of detergents, adhesives and new polymeric foams [20]. The *Foam2Foam* project is developing technologies for the chemical recycling of generic PFW, to produce green polyols as a raw material in the manufacturing of new polyurethane foams [21]. Finally, the *RECALZA* project has developed investigations to close the life cycle of the polyurethane foams used in the manufacture of footware through chemical recycling by glycolysis [22].

Numerous investigations have likewise been carried out for the design of new construction materials based on polyurethane waste. Its properties mean that polyurethane may be incorporated in new materials with good thermal and acoustic performance. Some examples are the manufacture of concrete, and cement mortars with rigid polyurethane foams from the refrigeration chamber-panel-making industry, and for the automobile industry [23,24,25,26,27,28,29]. Moreover, the studies completed on the use of gels obtained from PFW for soil stabilization [30] and the manufacture of elastomers and geopolymers are also of great interest [31,32].

There are investigations oriented towards the design of improved gypsum mortars through the inclusion of rigid polyurethane foam waste [33,34,35,36]. Nevertheless, no references have been found to prefabricated construction materials that integrate polyurethane waste The *Life Repolyuse* project proposed, to good effect, the manufacture of gypsum panels with polyurethane waste, for their use in the manufacture of technical roofs [19].

In this work, the design of gypsum mortars fabricated with rigid PFW is reported for their use either as conglomerate materials or as raw materials for the manufacture of prefabricated construction products.

## 2. Materials and Methods

The objective of this investigation is the design and characterization of gypsum mortars for the manufacture of Interior Thermal Insulation Systems (ITIC) panels, with the objective of improving the energetic behaviour of buildings.

Various mixtures of gypsum were designed with the added value of PFW, characterizing their properties in both the fresh and the hardened state.

### 2.1. Raw Materials

The gypsum mortars were designed with two types of binders, together with variable quantities of recovered PFW. Subsequently, standardized sample specimens were cast in moulds, in preparation for the characterization tests and to study the effects of those additions on the physical–mechanical properties of the resulting mixtures, in accordance whit standard EN 13279-2 [37]. The following materials were used:

- Gypsum binder Type A1, as per EN 13279-1 [38], with a density of 816 kg/m^3^. The binder consisted of calcium sulphate in its different phases of hydration (calcium sulphate hemihydrate (*CaSO*_4_·0.5 *H*_2_*O*) and anhydrite (*CaSO*_4_)), obtained from partial dehydration of the calcium sulphate dihydrate (*CaSO*_4_·2*H*_2_*O*) phase;
- Gypsum binder Type B1, according to EN 13279-1 [38], with a density of 1035.8 kg/m^3^. The binder consisted of gypsum with a minimum of 50% calcium sulphate as the principal active component and a lime content (calcium hydroxide (*Ca*(*OH*)_2_)) below 5%;

The two gypsum binders were manufactured and supplied by the firm Yesos ALBI (Grupo CALCINOR), from its factory at Villalómez (Burgos, Spain). Their technical characteristics are shown in Table 1.

**Figure 1:**
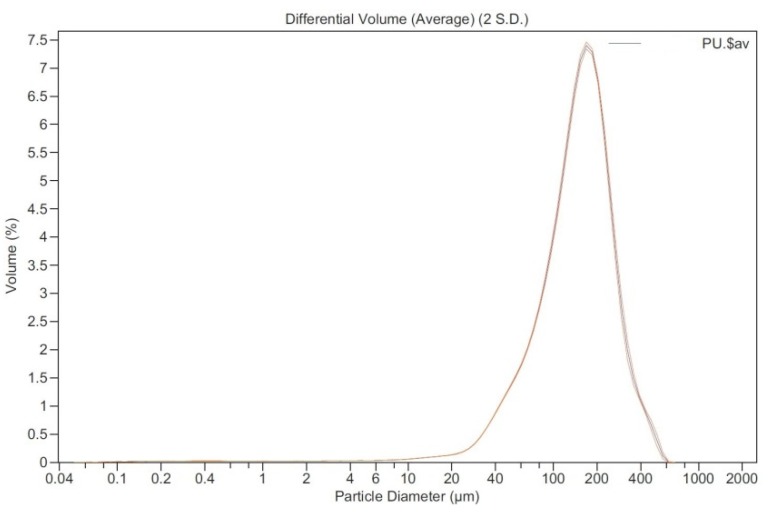
Particle size distribution of polyurethane foam waste (PFW).

- Polyurethane Foam Waste (PFW), with a density of 90,687 kg/m^3^, was supplied by the firm Kingspan Prax, S.A. (Burgos, Spain). The waste product is from a factory cutting process, in which large-sized blocks of polyurethane are sawn to the sizes required by the manufacturer. This *polyurethane dust* is recovered directly from the sawing mill by a vacuum coupled to a sleeve filter where the waste accumulates. Distribution by grain sizes, represented in Figure 1, was studied using a Beckam Coulter LS.13.320 Particle-Size Analyzer.

The chemical composition of the polyurethane foam was determined by means of elemental analysis, a LECO CHNS-932 Elementary Chemical Analyzer. The results are shown in Table 2.

Water: from the urban mains supply, acceptable for use in the manufacture of mortars.

**Figure 2:**
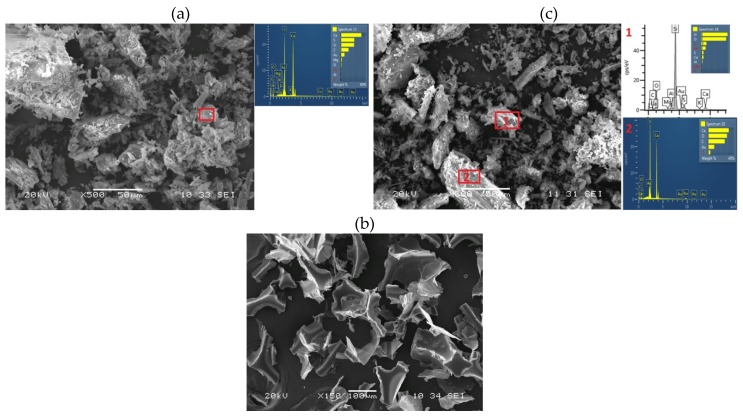
Gypsum binder Type A1 (**a**); Gypsum binder Type B1 (**c**); PFW (**b**).

The three raw materials employed for the manufacture of the mortars was analyzed with a model JEOL JSM-6460-LV Scanning Electron Microscope (SEM) (JEOL, Ltd. Akishima, Tokyo-Japan), equipped with an elemental INCA analysis system, with an acceleration voltage of 0.20–30 KV. The samples had previously been coated by low-vacuum cathode sputtering (gold) with an EMITECH k550X sputter coater, at a current of 30 mA over 2 min in an argon atmosphere. The SEM images obtained are shown in Figure 2. In binder Type B1, the presence of mineral aggregates alongside the hemihydrate was confirmed. It may be assumed from the results of the elemental analysis that the aggregates were of a silica nature.

### 2.2. Mortar Mixtures

Gypsum mortars have been designed from two types of binders: A1 and B1, together with different amounts of PFW, by volume.

In Table 3, the labels of each mix are shown, as well as the ratio of components by weight. In addition, reference mortars (no foam) were prepared, in order to compare the results with the mixtures that were designed.

Except for binder Type A1, the other gypsums were considered pre-mixed gypsums, as per the specifications in standard EN 13279-1 [38], for which reason the water/gypsum ratio was determined with the *flow table* method. The w/b (water/binder) ratio of the reference mortar, A1, was also determined by the same method.

In accordance with these criteria, the necessary amount of water was added to the mixtures, to yield a diameter on the flow table of 160 ± 5 mm, as per the specifications of standard EN 13279-2 [37]. The gypsum binder was first of all dry-mixed with the polyurethane waste, for the preparation of the mortars. Water was then added to the gypsum and the PFW and the mixes were manually stirred with a spatula for 1 min. Subsequently, it was mixed in a Proetic C0087 planetary mixer for another minute.

### 2.3. Mortar Characterization

#### 2.3.1. Characterization in Fresh State

Mortar density in the fresh state was calculated as per standard UNE 102042 [39], by difference un weight in a recipient of known volume, first full of mortar and then empty. The *Vicat Cone* test procedure was followed to determine the Time to Onset of Setting [38]. A rubber mould placed on a plate was filled with gypsum mortar. A Φ 10 mm plunger weighing 100 g (Vicat apparatus) was used and the time taken by the needle at the end of the plunger to penetrate to a depth of 22 ± 2 mm down onto the glass plate was noted.

#### 2.3.2. Characterization in Hardened State

Three standardized specimens 40 × 40 × 160 mm^3^ were prepared for each dosage, to perform the flexural and compressive tests. The specimens were demoulded on the following day and remained in the laboratory for seven days, at a temperature of 23 ± 5 °C and at a relative humidity of 50% ± 5%.

Prior to the completion of the tests, the specimens had been dried in an oven at a temperature of 40 °C, until they reached a constant mass. On the following day, the specimens were removed from the oven and introduced in a drier, until their temperature fell to the same level as the temperature in the laboratory, and the dry weight of the three specimens was determined, to obtain the dry bulk density.

**Figure 3:**
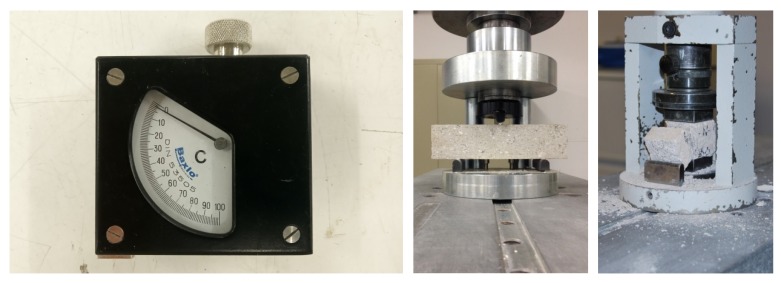
Shore C Durometer C (left); Flexural test (centre); Compressive test (right).

A Shore durometer with a sensitivity of 1 Shore unit was employed, in order to measure the surface harness or Shore hardness of the specimens (Figure 3). Five measurements were performed on each of the longitudinal sides corresponding to the sides of the mould in each specimen.

Having determined the mortar density and its surface strength, the specimens were then broken in a Supercar MEM-101/SDC hydraulic press of 200 kN. A load centred mid-span on the specimens of hardened mortar was applied up until failure, in order to determine their flexural strength. The separation between supports was 100 mm (Figure 3). The two fragments obtained from the breakage under flexion were tested under compression, on a 40 × 40 mm^2^ surface, applying a constant load and with no accelerations until its breakage, in accordance with the specifications of standard EN 13279-2 [37].

A 10 mm layer of gypsum was applied to a ceramic brick, previously submerged in water for 1 min, to avoid loss of mix-water from the mixtures. Before starting the setting process, the circular surfaces of a diameter of 50 mm were drawn on the gypsum layer. The samples remained for 7 d in the laboratory at a temperature of 23 ± 5 °C and at a relative humidity of 50% ± 5%. 

Following 7 d of curing and hardening, metallic pads were adhered to the samples with a bi-component glue to apply traction force. A traction test apparatus applied a perpendicular load to the test areas through the metallic pads. The load was applied without shaking and at a uniform speed [38].

The procedure from standard EN 1015-19 [40] was used to establish the Water Vapour Permeability coefficient. To do so, cylindrical specimens of a diameter of 160 mm and a height of 16 mm were moulded. After 7 d of curing had elapsed, they were placed in test recipients with a solution of potassium nitrate (KNO_3_), with the objective of maintaining a constant internal water-vapour pressure, different from the exterior atmosphere. Subsequently, the sides were sealed with a waterproof tape and kept in an environment at 20 ºC and at a relative humidity of 50%. Then, the recipients were weighed at appropriate intervals of time, to determine the flow of water vapour through the gypsum mortar.

As the properties of gypsum mortar material are affected by humidity, specimens with dimensions of 40 × 40 × 160 mm^3^ were moulded and the absorption of water by capillarity was determined by following the procedure from standard EN 1015-18 [41]. To do so, the specimens were arranged in a vertical position within an open cage placed in a tray, in such a way that the ends of the specimen were not in contact with the bottom of the tray, and they were then submerged in water to a height ranging from 5 to 10 mm. After 10 min, they were extracted from the water and weighed to obtain the coefficient of water absorption by capillarity. In addition, the height reached by the water was noted.

Finally, total water absorption of the mortars was determined by submerging the 40 × 40 × 160 mm^3^ specimens in a recipient until their saturation, to obtain their saturated weights.

## 3. Results and Discussion

### 3.1. Characterization in the Fresh State

The results of the in-fresh mortar characterization tests designed in the fresh state are shown in Table 4.

As may be observed, both in the mortars containing gypsum Type A1 and in the mortars with gypsum Type B1, the higher the amount of added polymeric waste, the higher the amount of water, because of the high water-absorption capacity of the PFW [27]. This behaviour can produce a significant increase in mixture porosity, as well as a reduction in their mechanical strengths.

Likewise, their in-fresh density was higher in the B1 mortars when compared with the A1 mortars. This behaviour may be explained by the mineral aggregates that accompany the Type B1 binder, as specified in standard EN 13279-1 [37]. A behaviour that is not possible in the Type A1 binder, composed in its totality of semi-hydrated calcium sulphate.

**Figure 4:**
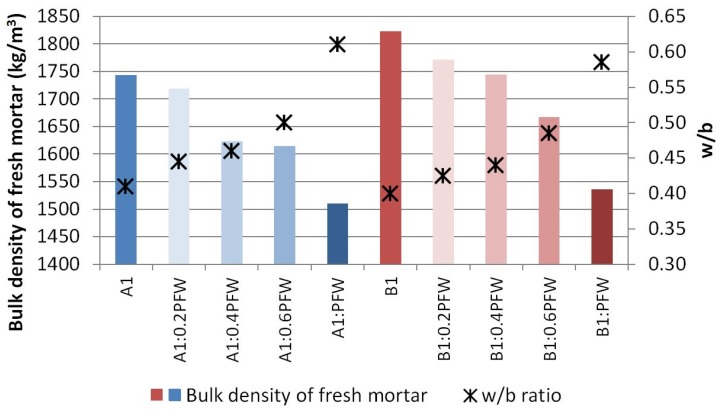
Bulk density of fresh mortar vs. w/b ratio.

The reduction in mortar density is related to the quantity of water added to the mixtures, as may be observed from Figure 4. Likewise, as the water/binder ratio increased, the density fell. This behaviour was similar in both the mortars dosed with binder type A1 and binder type B1, and both results are in agreement with those of earlier investigations [34].

In relation to the onset of setting time test, the mortars dosed with binder Type A1 and PFW, as may be seen from Table 4, showed a tendency to lengthen their time to onset of setting, as the amount of waste was progressively increased. In the case of the mortars made with binder Type B1, the samples dosed with proportions of 0.2, 0.4 and 0.6 of PFW presented times to onset of setting that were similar to reference mortar B1, although with a clear difference in the case of mortar B1:PFW.

When the analysis was completed as a function of the binder that was employed, it was observed that the times to onset of setting of the mortars manufactured with binder Type B1 were lower with respect to their counterparts dosed with binder Type A1. This behaviour can be explained by the presence in binder Type B1 of mineral aggregates and dihydrates that function as a nucleus of crystallization. These impurities contribute to more rapid crystal formation in the hydration process than in the case of binder Type A1, because the presence of Anhydrites I and II are frequent, on account of their greater purity, which slows the reaction process of water with the material [42].

### 3.2. Characterization in Hardened State

The mechanical characterization test results are shown in Table 5.

As may be observed, the incorporation of PFW in the mixtures led to a progressive reduction in density in the hardened state, which was higher as larger quantities of foam were added, showing a similar behaviour to mortars in the fresh state. 

**Figure 5:**
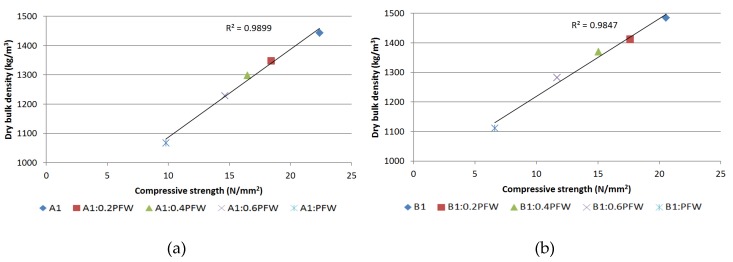
Compressive strength vs. dry bulk density. Type A1 (**a**); Type B1 (**b**).

The reduction in the density of the hardened mortars as larger amounts of PFW were added likewise reduced the mechanical strengths (flexural, compressive and adherence), as may be observed in Figure 5, with a special impact on the mixtures that incorporated higher concentrations of PFW [34].

According to the data in Table 5, a first conclusion was that, in all cases, the mortars dosed with binder type A1 showed higher mechanical strengths, both flexural and compressive, with regard to the mortars dosed with binder type B1. 

When the loss of mechanical strength, due to the incorporation of maximum amounts of PWF, was analysed, the flexural strength of mortar A1:PFW was reduced with regard to its reference mortar, A1, by 54.5% and its compressive strength was reduced by 56.2%. In the case of mortar B1:PFW with regard to B1, the flexural strength was reduced by 65.8%, and its compressive strength by 67.8%.

In accordance with this criterion, when the amount of PWF added to the mixtures was 0.6, the reduction in their mechanical strength was approximately 40%, so that amount is considered the optimal option for the quantity of waste/mechanical strength ratio. In this way, mortars A1:0.6PFW and B1:0.6PFW were selected for detailed study in a second stage of the investigation.

Nevertheless, the strengths of all the design mortars surpassed the minimum references established in standard EN 13279-2 [37], regardless of the large quantity of PFW added to the mixtures, i.e., a minimum flexural strength of 1.0 MPa and a minimum compressive strength of 2.0 MPa.

**Figure 6:**
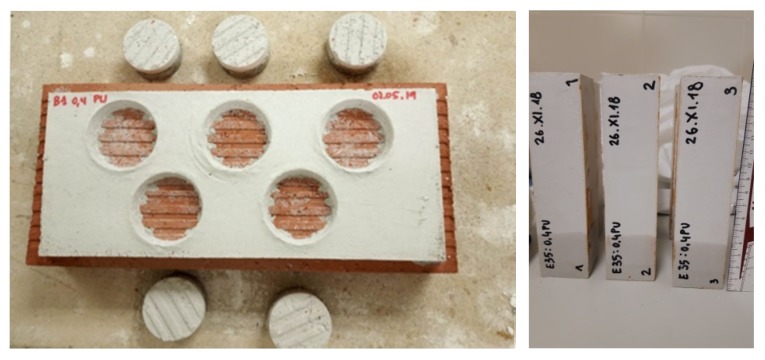
Adherence test (left), height of water after 10 min of the suction test (right)

On the one hand, in the case of adherence strength, greater strengths were likewise obtained in the mortars dosed with binder type A1, while the mortars dosed with binder type B1 recorded lower values in all cases. On the other hand, as can be seen from Table 5, the adherence strength fell as the amount of PWF added to the mixtures increased and, at the same time, the quantity of water increased. However, the differences that were observed were not significant and the results were encouraging, because, in all cases, breakage under traction occurred within the mortar–brick interface (adherence failure) (Figure 6).

With regard to Shore C hardness, the values were quite uniform in all cases (>90), except for the mortars in which the amount of PFW was greater (A1:PFW and B1:PFW). 

Gypsum materials are sensitive to humidity. As observed, the incorporation of PFW in the mixtures increased the amount of mix water and, in consequence, their porosity. Capillary Water Absorption, Total Water Absorption, and Water-Vapour Permeability were determined, to establish the behaviour of the mortar designs against the action of water. The test results are shown in Table 6.

Taking the Coefficient of Water Vapour Diffusion (µ) as a reference, the lower the value, the lower the resistance of the mortar to the passage of water vapour through its internal pores. All the mortars under study were shown to have very low coefficients (µ ≤ 7), and so they all presented a minimal risk of interstitial condensation within their interior.

It is worth pointing out that the mortars with larger amounts of PWF, A1:PFW and B1:PFW, presented the lowest values: µ = 3. 

With regard to capillarity water absorption, the mortars presented high coefficients, corresponding to gypsum mortars, and, in consequence, the water penetration height within the mortar was very high. It was observed that the water penetration height in the mortars was greater when binder type A1 was employed. This result implied that the consolidation of their interstitial capillary network was also greater, due to the purity of the material.

When the study was completed it was observed that, as a function of the amount of PFW added to the mixtures, the water reached higher levels as larger amounts of PWF were added.

Likewise, in the Total Water Absorption Test, the mixtures made with binder type A1 had higher levels than those dosed with B1. This result implies that the *network of open pores* was greater in mixtures A1, so they presented lower µ values and were more permeable to the water vapour passing through their pores. In addition, the test results of the suction test, to determine the height reached by the water, led us to assume that a fine capillary network with many interconnected pores formed, for which reason the water rose easily, due to the rising forces of capillarity [43].

## 4. Conclusions

The characterization tests of the gypsum mortars dosed with PFW have clarified how the dosing of PFW influences the mortar properties. 

The first general conclusion from the experimental process leads us to affirm that the use of PFW in the manufacture of gypsum mortars determines an important change in the properties with regard to the reference mortars:

- The incorporation of PFW significantly reduced the density of the gypsum mortars, both in the fresh and in the hardened state, increasing the water/gypsum ratio in the mixtures, due to the hygroscopic nature of PFW;
- The gypsum mortars reduced their mechanical strength under both flexural and compressive as larger quantities of PFW were incorporated. Nevertheless, in all cases, the gypsum mortar designs with PFW showed good mechanical properties for use as a construction material;
- With regard to their adherence, the incorporation of PFW in the mixtures also reduced adherence to the ceramic substrate, but not significatively, yielding acceptable values for a rendering material;
- Despite the fact that PFW is a soft and a flexible material, the Shore C surface hardness was maintained at acceptable values in all the mixtures, in comparison with the reference mortars, A1 and B1, where it was lower whenever larger dosages of PFW were incorporated;
- The mortars dosed with PFW presented higher water vapor diffusion levels. In addition, the greater the amount of added foam waste, the greater the water absorption by capillarity.

## Figures and Tables

**Table 1:** Properties of the gypsum conglomerates (Yesos Albi S.A.).

	A-1	B-1
Granulometry	0–2 mm	0–2 mm
Purity index	>92.0%	—
Content in CaSO_4_	>87.0%	>76.0%
Bending strength	>1.5 N/mm^2^	>2.0 N/mm^2^
Compressive strength	>3.5 N/mm^2^	>3.0 N/mm^2^
Water/Gypsum ratio	0.66	0.40
Onset of setting	>15 min	>20 min

**Table 2:** Chemical composition of Polyurethane Foam Waste (PFW).

	P (mg)	N (%)	C (%)	H (%)	S (%)
PFW	1.170	7.46	53.94	4.74	0.0

**Table 3:** Mortar mixtures.

Sample		Volume			Weigh (g)		
	A1	B1	PFW	A1	B1	PFW	Water
A1	1	—	—	1000	—	—	410.00
A1:0.2PFW	1	—	0.2	1000	—	22.23	454.89
A1:0.4PFW	1	—	0.4	1000	—	44.45	480.45
A1:0.6PFW	1	—	0.6	1000	—	66.68	533.34
A1:PFW	1	—	1	1000	—	111.14	677.80
B1	—	1	—	—	1000	—	400.00
B1:0.2PFW	—	1	0.2	—	1000	17.51	432.44
B1:0.4PFW	—	1	0.4	—	1000	35.02	455.41
B1:0.6PFW	—	1	0.6	—	1000	52.53	510.48
B1:PFW	—	1	1	—	1000	87.55	636.22

**Table 4:** Characterization in the fresh state.

Sample	w/b	Density (kg/m^3^)	Onset of Setting Time (min:sec)	Onset of Setting Time EN 13279-2:2014 (min)
A1	0.410	1744	10:40	11
A1:0.2PFW	0.445	1719	09:33	10
A1:0.4PFW	0.460	1623	10:50	11
A1:0.6PFW	0.500	1615	12:10	12
A1:PFW	0.610	1510	12:25	12
B1	0.400	1823	06:00	6
B1:0.2PFW	0.425	1771	07:00	7
B1:0.4PFW	0.440	1745	06:30	7
B1:0.6PFW	0.485	1667	07:30	8
B1:PFW	0.585	1536	09:45	10

**Table 5:** Mechanical properties.

Sample	Dry Bulk Density (kg/m^3^)	Flexural Strength (N/mm^2^)	Compressive Strength (N/mm^2^)	Adherence Strength (N/mm^2^)	Shore Hardness (Shore C)
A1	1443	8.48	22.38	1.08	93
A1:0.2PFW	1348	6.80	18.42	0.82	92
A1:0.4PFW	1299	5.82	16.47	0.72	92
A1:0.6PFW	1229	4.68	14.62	0.68	90
A1:PFW	1068	3.86	9.80	0.66	81
B1	1485	7.70	20.54	1.05	95
B1:0.2PFW	1413	6.09	17.62	0.79	93
B1:0.4PFW	1371	5.59	15.04	0.59	92
B1:0.6PFW	1283	4.26	11.67	0.51	91
B1:PFW	1112	2.63	6.61	0.46	79

**Table 6:** Mortar behaviour in the presence of water.

Sample	Water Vapour Permeability		Water Absorption Due to Capillary Action		Total Water Absorption (%)
	(kg/m·s·Pa)	µ	Coefficient (kg/m^2^·min^0.5^)	Height (mm)*	
A1	3.7975 × 10^−11^	5	2.73	37	20.86
A1:0.2PFW	3.4754 × 10^−11^	5	2.79	44	23.11
A1:0.4PFW	4.3428 × 10^−11^	5	3.07	48	25.62
A1:0.6PFW	4.8993 × 10^−11^	4	3.33	49	29.34
A1:PFW	6.7349 × 10^−11^	3	3.80	56	38.99
B1	2.8367 × 10^−11^	7	2.61	24	15.00
B1:0.2PFW	2.8570 × 10^−11^	7	2.64	28	16.58
B1:0.4PFW	2.7536 × 10^−11^	7	2.71	29	18.79
B1:0.6PFW	3.3085 × 10^−11^	6	3.09	34	23.53
B1:PFW	6.3198 × 10^−11^	3	3.89	43	32.38

* Water height at 10 min.
